# Towards a sole β‐amyloid‐centered definition of Alzheimer’s disease? Risks and alternatives

**DOI:** 10.1002/alz.089096

**Published:** 2025-01-09

**Authors:** Nicolas Villain, Bernard J Hanseeuw, Vincent Planche

**Affiliations:** ^1^ Sorbonne Université, INSERM U1127, CNRS 7225, Paris France; ^2^ Assistance Publique Hopitaux de Paris (APHP), Paris France; ^3^ Institute of Neuroscience ‐ UCLouvain, Brussels Belgium; ^4^ Massachusetts General Hospital, Harvard Medical School, Boston, MA USA; ^5^ Centre Mémoire de Ressources et de Recherches, Pôle de Neurosciences Cliniques, CHU de Bordeaux, Bordeaux France; ^6^ Institut des Maladies Neurodégénératives, Bordeaux France

## Abstract

**Aims:**

The Alzheimer Association (AA) has proposed new diagnostic criteria for Alzheimer’s disease (AD) based on biomarkers coinciding with β‐amyloidosis onset. However, there are concerns regarding the implications of these criteria.

**Methods:**

We reviewed several perspectives, including disease definition, public health, philosophy, therapeutic, and diagnostic.

**Results:**

1. Disease definition – the AA definition displays limited and heterogeneous predictive capabilities of lifetime clinical progression in asymptomatic individuals, and a different disease label may be used, as seen in other examples in medicine (eg MGUS and multiple myeloma).

2. Public health – the majority of the A+ population is asymptomatic, and the AA definition may reduce the association between the “label” AD and dementia or subsequent progression to dementia, which could impact public health decisions.

3. Philosophy – the clinical and biological definitions of AD are based on different medical‐philosophical theories of disease (Nordenfelt vs. Boorse), and if the AA criteria are widely adopted, there may be misconstrued perceptions among stakeholders.

4. Therapeutic – an amyloid definition of AD disconnects the biological endpoint from the clinically relevant outcome, which has led to the approval of aducanumab without demonstrating clinical impact. This raises concerns about future decisions regarding asymptomatic individuals.

5. Diagnostic – the AA framework lacks clear guidelines on linking clinical situations with AD biological changes, which may result in misdiagnosis and overdiagnosis in patients with abnormal biomarkers.

**Conclusions:**

Defining AD based solely on β‐amyloidosis is risky and reduces association with clinically relevant outcomes. Diagnosing AD should instead involve symptoms or a high probability of developing them. The IWG framework takes a cautious approach, focusing on symptom onset. Future IWG developments could expand the “preclinical AD” label by integrating specific biomarkers that can predict severe cognitive decline in asymptomatic patients.

